# Combined Expression Trait Correlations and Expression Quantitative Trait Locus Mapping

**DOI:** 10.1371/journal.pgen.0020006

**Published:** 2006-01-20

**Authors:** Hong Lan, Meng Chen, Jessica B Flowers, Brian S Yandell, Donnie S Stapleton, Christine M Mata, Eric Ton-Keen Mui, Matthew T Flowers, Kathryn L Schueler, Kenneth F Manly, Robert W Williams, Christina Kendziorski, Alan D Attie

**Affiliations:** 1 Department of Biochemistry, University of Wisconsin, Madison, Wisconsin, United States of America; 2 Department of Statistics, University of Wisconsin, Madison, Wisconsin, United States of America; 3 Department of Nutritional Sciences, University of Wisconsin, Madison, Wisconsin, United States of America; 4 Department of Horticulture, University of Wisconsin, Madison, Wisconsin, United States of America; 5 Departments of Anatomy and Neurobiology, University of Tennessee Health Science Center, Memphis, Tennessee, United States of America; 6 Department of Biostatistics and Medical Informatics, University of Wisconsin, Madison, Wisconsin, United States of America; North Carolina State University, United States of America

## Abstract

Coordinated regulation of gene expression levels across a series of experimental conditions provides valuable information about the functions of correlated transcripts. The consideration of gene expression correlation over a time or tissue dimension has proved valuable in predicting gene function. Here, we consider correlations over a genetic dimension. In addition to identifying coregulated genes, the genetic dimension also supplies us with information about the genomic locations of putative regulatory loci. We calculated correlations among approximately 45,000 expression traits derived from 60 individuals in an F_2_ sample segregating for obesity and diabetes. By combining the correlation results with linkage mapping information, we were able to identify regulatory networks, make functional predictions for uncharacterized genes, and characterize novel members of known pathways. We found evidence of coordinate regulation of 174 G protein–coupled receptor protein signaling pathway expression traits. Of the 174 traits, 50 had their major LOD peak within 10 cM of a locus on Chromosome 2, and 81 others had a secondary peak in this region. We also characterized a Riken cDNA clone that showed strong correlation with stearoyl-CoA desaturase 1 expression. Experimental validation confirmed that this clone is involved in the regulation of lipid metabolism. We conclude that trait correlation combined with linkage mapping can reveal regulatory networks that would otherwise be missed if we studied only mRNA traits with statistically significant linkages in this small cross. The combined analysis is more sensitive compared with linkage mapping alone.

## Synopsis

In order to annotate gene function and identify potential members of regulatory networks, the authors explore correlation of expression profiles across a genetic dimension, namely genotypes segregating in a panel of 60 F_2_ mice derived from a cross used to explore diabetes in obese mice. They first identified 6,016 seed transcripts for which they observe that the gene expression is linked to a particular region of the genome. Then they searched for transcripts whose expression is highly correlated with the seed transcripts and tested for enrichment of common biological functions among the lists of correlated transcripts. They found and explored the properties of 1,341 sets of transcripts that share a particular “gene ontology” term. Thirty-eight seeds in the G protein–coupled receptor protein signaling pathway were correlated with 174 transcripts, all of which are also annotated as G protein–coupled receptor protein signaling pathway and 131 of which share a regulatory locus on Chromosome 2. The authors note many of these findings would have been missed by simple expression quantitative trait loci analysis without the correlation step. The approach was used to identify a common set of genes involved in lipid metabolism.

## Introduction

Biological systems are tightly controlled such that the concentrations of components are precisely balanced in an active biological pathway. The balances are maintained in part by coordinately regulated expression levels of the genes involved in the biological processes. Thus, genes that are coregulated across various physiologic conditions are likely to have similar functions. This creates a context by which the function of unannotated genes can be predicted.

One of the greatest challenges to biologists today is to functionally characterize genes identified by the genome sequencing projects. Traditionally, gene functions are inferred based on similarities in primary sequences, functional domains, or structures of encoded proteins [1]. In contrast to these qualitative approaches, we and others are using quantitative methods to predict gene function and discover gene regulatory networks [2,3].

Eisen and colleagues [4] were among the first to use microarray gene expression profiles for gene function prediction. They used hierarchical clustering algorithms to analyze time-course gene expression profiles of budding yeast and human fibroblasts and found that grouping of transcripts with similar profiles across a time dimension could accurately identify genes of similar function. Recently, Zhang and colleagues [3] generated microarray expression data for nearly 40,000 mRNAs in 55 mouse tissues and found that coordinated expression across a tissue dimension can contribute to the prediction of gene function. Although extremely informative, these approaches do not establish lines of causation among correlated mRNAs and therefore cannot provide the origin of a particular gene regulatory network.

Genetics establishes a one-way line of causation between genotype and phenotype. Brem et al. [5] used microarray data as phenotypes for genetic mapping in a segregating yeast sample. This approach has inspired the mapping of expression quantitative trait loci (eQTL) in mammalian species [6–12]. Each study identified *cis* and *trans* traits. A *cis* trait is one that genetically maps to the physical location of the gene encoding its mRNA, suggesting that variation at the locus is responsible for the heritable changes in gene expression. A *trans* trait maps to a region distinct from its physical location and thus implies the location of a potential regulator acting in *trans*. Theoretically, when multiple traits map together in *trans* (i.e., overlapping eQTL peaks within an approximately 10-cM region), we can hypothesize that they are coregulated by a common regulator encoded by a gene locus underlying the eQTL. Unfortunately, in practice, because mRNAs are typically regulated by multiple factors, individual *trans*-acting eQTL exert a relatively weak genetic signal and are difficult to detect in a small sample size. This limits the power to detect metabolic regulators.

Schadt et al. [9] surveyed 23,574 genes using Rosetta oligonucleotide arrays. More recent studies used Affymetrix U74A arrays, or RAE230A arrays, which covered about one third of all the transcripts [7,8,11]. The transcripts chosen for measurement were biased toward known genes. Here, we measured the expression levels of the transcripts on the Affymetrix MOE430 Set arrays, which include many genes of unknown function.

We studied an F_2_ segregating for obesity and diabetes to expose key components of gene regulatory networks. To this end, we combined correlation analysis with linkage mapping. Rather than considering correlation across a time or tissue dimension, we evaluated correlation across a genetic dimension, as created by a segregating F_2_ sample. An F_2_ represents an allelic block permutation. Our combined approach increases the power to identify metabolic regulatory networks.

## Results

We generated an F_2_-*ob/ob* sample from the C57BL/6J (B6) and BTBR founder strains. The B6 and BTBR strains, when made obese, differ in diabetes susceptibility [13]; B6-*ob/ob* mice are diabetes resistant, whereas BTBR-*ob/ob* mice develop severe diabetes. The F_2_ mice were genotyped for 194 markers (average marker interval of approximately 10 cM). A subset of 60 mice was chosen using a selective phenotyping algorithm developed in our group [14]. From each liver, 45,037 expression measurements were obtained using the Affymetrix MOE430 microarrays. According to the December 2004 gene annotation table, 14,487 (32.2%) of the transcripts have unique RefSeq protein identifiers, and 16,466 (36.6%) have been annotated to at least one Gene Ontology (GO) Biological Process (BP). Altogether, about two thirds of the transcripts that we measured were not annotated in either of these two sources.

### Mapping of Metabolic Regulatory Loci

After processing the Affymetrix CEL files with Robust Multi-array Average (RMA) [15], we used interval mapping to screen the 45,037 traits. Transcripts with LOD scores of 3.4 or greater at one or more locations in the genome were recorded as mapping transcripts. Use of this LOD threshold roughly corresponds to a genomewide type I error rate of 0.08 [16] and a false discovery rate (FDR) of 0.48 (see Materials and Methods). The high FDR is not surprising, considering that adjustments for multiple tests across transcripts have not been made [17]. We nevertheless keep this LOD threshold since we consider this step as only an initial screen; the threshold reduces the computational burden significantly while maintaining a high true-positive rate. This procedure yielded 6,016 mapping transcripts.

**Figure 1:**
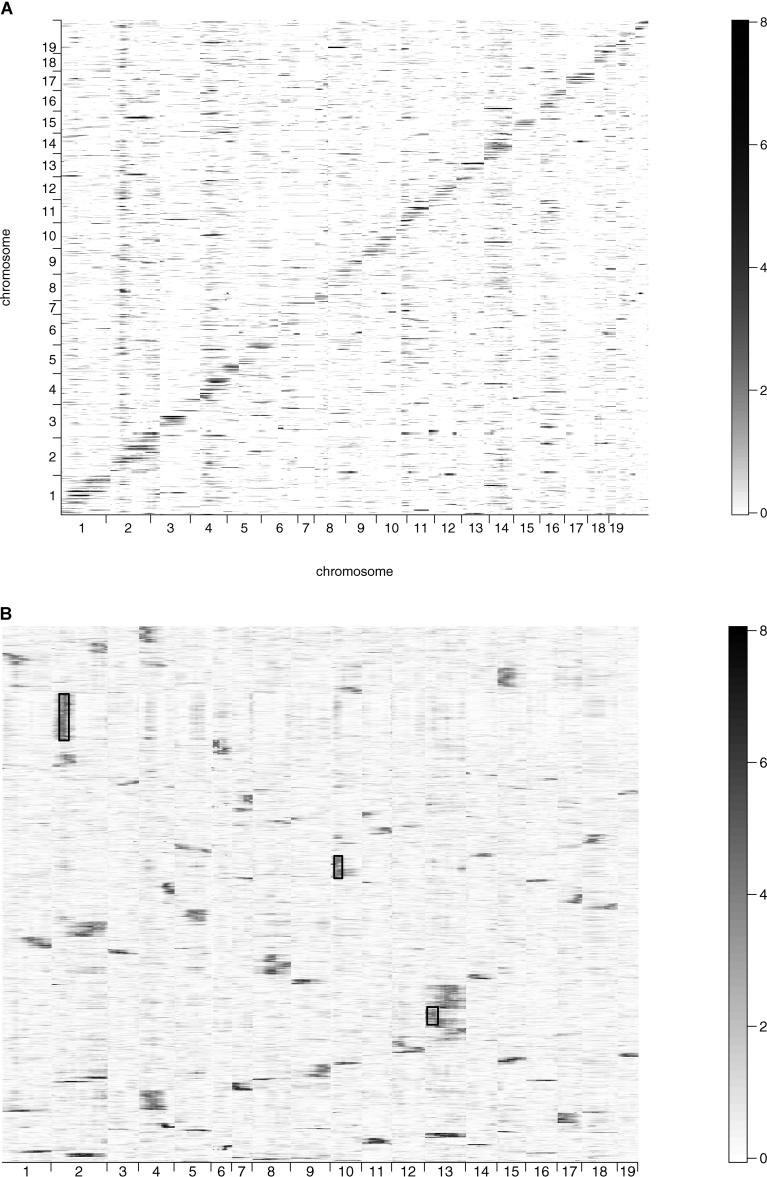
Expression QTL in the (B6 × BTBR) F_2_-*ob/ob* Cross (A) Expression QTL that regulate gene expression in the (B6 × BTBR) F_2_-*ob/ob* cross. The physical locations of the transcripts are organized on the y-axis. The chromosome regions (x-axis) to which those transcripts are mapped were obtained using the *scanone* function in the R/qtl package with 5-cM intervals. The gray scale reflects the strength of the linkage signals (LOD scores greater than 8 are scaled to 8). Transcripts appearing on the diagonal are inferred to be *cis*-regulated. The chromosomes were concatenated to form a 2,300-Mb genome, starting from Chromosome 1 and ending with Chromosome 19. (B) Global display of mapping patterns of linkage clusters. The y-axis shows the 6,016 mapping transcripts. The x-axis shows the physical locations to which these transcripts map. The transcripts are grouped based on the hierarchical clustering of linkage mapping patterns across the genome. Darker areas indicate the regions to which traits are comapped or coregulated. The boxes correspond to the hot spots on Chromosomes 2, 10, and 13. The gray scale reflects the strength of the linkage signals (LOD scores greater than 8 are scaled to 8).

We plotted the mapping locations of these expression traits against their physical locations (Figure 1A). Of the 6,016 mapping transcripts, 723 were classified as *cis* (the diagonal spots in Figure 1A) and the rest were classified as *trans.* The *cis* traits are listed in Table S1. Rearrangement of Figure 1A using hierarchical clustering of the LOD profiles enabled us to identify the putative locations of metabolic regulators (Figure 1B). We identified 15 regions with the highest number of mapping transcripts, and for each region, we tested the list of mapping traits for enrichment in a common functional annotation, using the GO database and a Hypergeometric calculation. For each region, we examined up to 50 GO terms, each of which involved more than ten genes. A region is said to be enriched for a particular GO term if the resulting Hypergeometric *P-*value is ≤0.01. Using these criteria, we found modest functional enrichment in each region (results not shown). However, when we followed up and examined all eQTLs that have similar maximum LOD positions (whether statistically significant or not), we found clear evidence of enrichment (Figure 3B). We performed the same procedure on randomized data, preserving the correlation structure among the traits. This enrichment test did not reveal results as significant as were observed in the original data.

**Figure 2:**
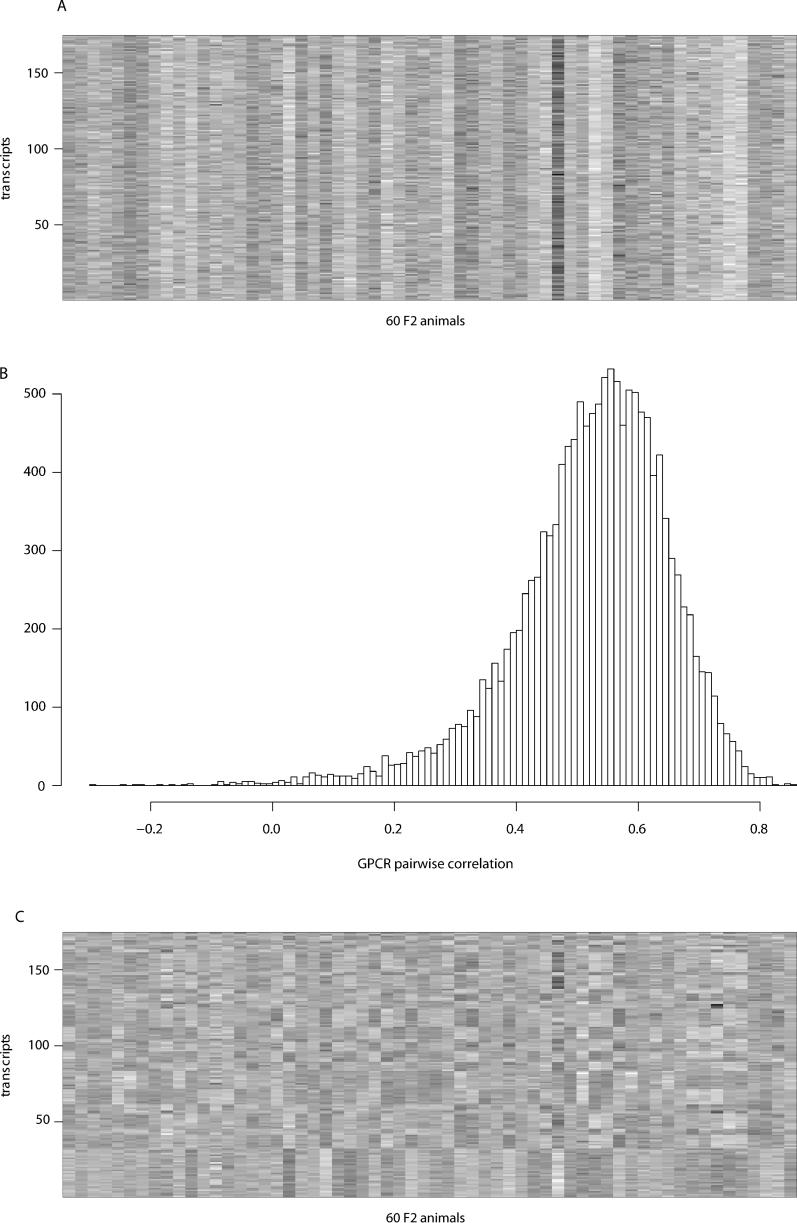
Coordinate Regulation of Genes for GPCR Protein Signaling Pathways (A) GPCR protein signaling pathway genes show coordinated changes across the 60 F_2_ mice. The rescaled expression levels of 174 distinct transcripts (y-axis) that belong to the GPCR pathway. The x-axis displays the 60 F_2_ mice. The plot shows that the subset of GPCR traits identified by our trait correlation-GO analysis has vertical patterns that indicate coordinate expression across the 60 F_2_ mice (*P* = 1e10^−4^ by permutation test). (B) Histogram of pairwise correlation coefficients of GPCR traits identified with different seeds. (C) Scaled expression levels of traits from one realization of the permuted lists. Thirty-eight lists of correlated traits were randomly sampled from a total of 1,341 correlated trait lists. The list sizes were adjusted to match those of the GPCR lists. The expression values were scaled to have mean intensity = 0 and variance = 1, as in the GPCR list in Figure 2A.

### Combining Mapping and Correlation Analysis Improves Sensitivity to Detect Regulatory Loci

We developed a strategy to explore metabolic regulatory networks on a genomewide scale that incorporates correlated traits that by themselves do not show statistically significant linkage. Considering each of the 6,016 mapping transcripts as a “seed,” we computed the Pearson correlation coefficients *(r)* across the 60 F_2_ mice for the 45,037 expression measurements and constructed a list of traits having *r* ≥ 0.7 (FDR = 1.52e10^−6^). We then tested for enrichment of any GO BP. Among the 6,016 seed transcripts, 1,341 produced lists of traits enriched for at least one GO term. The lists identified by the seed traits and GO terms are given in Table S2.

Among all of the lists from the 1,341 seed transcripts, we identified 862 unique GO BP terms (15,726 in total). We traced back the lists of correlated traits that are enriched for each unique GO term and concatenated all such lists. Different seeds may have identified overlapping traits enriched for the same GO term. These redundant copies of traits were removed after concatenation. By doing so, we associated a collection of distinct traits with each of the 862 unique GO terms. The full archive of lists is available in Table S3.

To illustrate how a gene mapping approach alone is insufficient, consider a locus on Chromosome 2 that regulates in *trans* multiple ion transport genes (Figure 1B, box), including six genes encoding subunits of the mitochondrial F_1_ or F_0_ ATPase of the proton channel of the electron transport system. It is known that there are 24 other ATP synthase subunits in the genome. None of the 24 expression traits exceeded the LOD threshold of 3.4, but many of them shared the linkage peak on Chromosome 2 (Figure S1). These results exposed a limitation of eQTL mapping; without inspection of the list of correlated *trans* traits, biological inference, and reconsideration of the 24 statistically insignificant LOD profiles, we would have missed evidence for coregulation of an entire family of mitochondrial respiratory genes. To automate this procedure, we consider augmenting eQTL mapping with trait correlation analysis.

**Figure 3:**
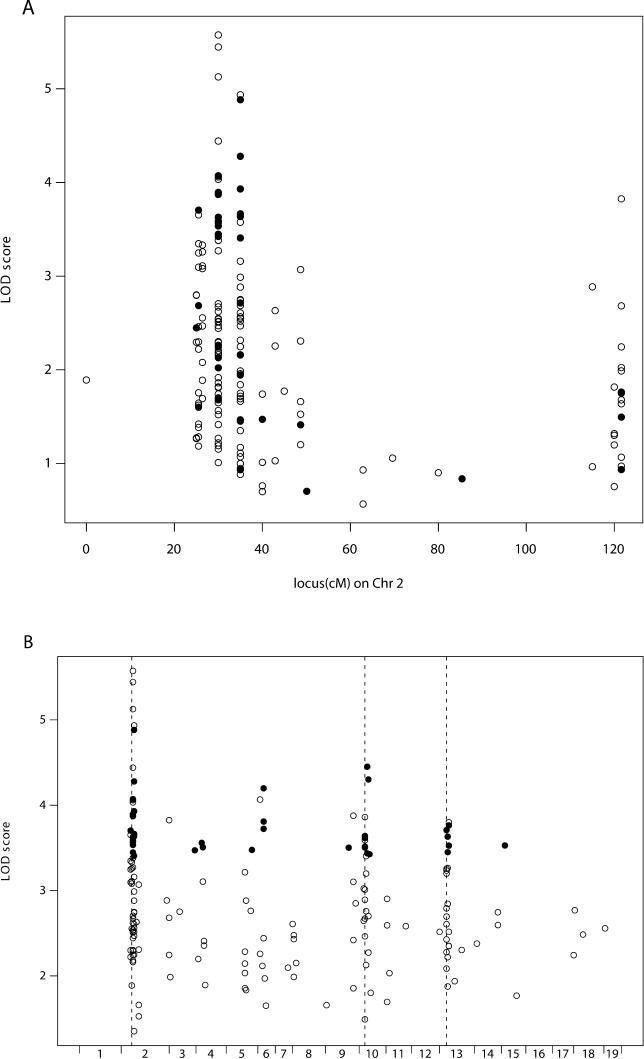
An Expression Quantitative Trait Locus on Chromosome 2 Regulates Many GPCR Protein Pathway Genes (A) Comapping of GPCR pathway traits to loci on Chromosome 2. The figure shows the peaks on Chromosome 2 versus LOD score for 174 GPCR protein signaling pathway traits. The closed circles correspond to 38 seed traits. (B) Comapping of GPCR pathway traits. The figure shows the magnitude (y-axis) versus location of LOD peaks (x-axis is scaled by cM) for 174 GPCR protein signaling pathway traits. The closed circles correspond to the 38 seed traits. The interval mapping LOD profiles for all 38 seeds are included in Figure S2.

### A Regulator Locus for G Protein–Coupled Receptor Genes

Using the correlation analysis described above, we identified 38 seeds that are in the G protein–coupled receptor (GPCR) protein signaling pathway and found 174 distinct traits correlated with these 38 seeds. Dramatically, all 174 traits were annotated as belonging to the GPCR protein signaling pathway GO term (Table S4). We created a heat plot to visualize the correlation patterns across multiple lists for this pathway by first rescaling the log_2_ intensity values of each trait across all the 60 F_2_ mice so that the mean = 0 and variance = 1. This rescaling step is necessary because some genes are overexpressed across all the F_2_ mice and others are underexpressed, which fails to reveal the coordinate regulation pattern across traits. We plotted the rescaled expression levels of the transcripts across the 60 F_2_ mice (Figure 2A). A striking feature of Figure 2A is the monochromatic columns, which reflect the coordinate changes in gene expression across the genetic dimension represented by the F_2_ sample. The traits from different seeds are not all highly correlated with each other (Figure 2B). However, the fact that they are enriched for the same GO term implies that they are coordinately regulated.

To ensure that the vertical patterns that were preserved across multiple lists were indeed functionally relevant, we randomly sampled 38 lists from the total of 1,341 lists and adjusted the sizes to be the same as those of the GPCR lists. We concatenated traits from those 38 randomly sampled lists. The vertical patterns shown in Figure 2A are no longer evident (Figure 2C). This was repeated 10,000 times. The vertical patterns in Figure 2A were shown to be statistically significant (*P* = 1e10^−4^; see Materials and Methods).

It is important to note that many of the traits correlated to seed traits did not show significant linkage across the 60 F_2_ mice and would have been overlooked by linkage mapping alone. When we mapped the GPCR seed with the most significant linkage, we discovered that the expression levels of this GPCR gene were influenced by a regulatory locus on Chromosome 2 at 30 cM, between *D2Mit297* and *D2Mit9* (Figure 3A). Of the 174 traits, 50 had their major LOD peak within 10 cM of this locus, and 81 others had a secondary peak in this region (Figure 3A). While many of these LOD scores do not exceed our significance threshold, the strong agreement suggests that most are driven by this same regulator or closely linked regulators, thus comprising a regulatory network. The LOD profiles for the 38 seed traits are shown in Figure S2. There appeared to be secondary regulators on Chromosomes 10 and 13 (Figure 3B). Similar evidence was suggested by inspecting the chromosome regions as shown in the boxes in Figure 1B. We picked out all transcripts, whether or not they have LOD scores exceeding 3.4, that share the same LOD peaks on the regions of Chromosome 2, 10, or 13 and tested for BP GO enrichment. We found GPCR protein signaling pathway in the enriched list with very small *P-*values. In short, we found that trait correlation combined with linkage mapping can reveal regulatory networks that would otherwise be missed if we studied only mRNA traits with statistically significant linkages in this small cross. The combined analysis is more sensitive compared with linkage mapping only.

### A Regulator Locus of Lipid Metabolism Genes

**Figure 4:**
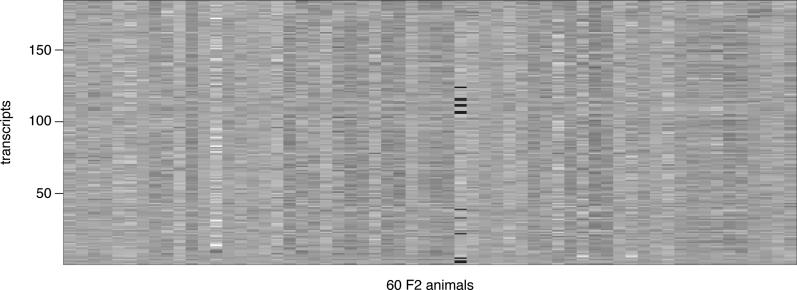
Lipid Metabolism Pathway Genes Show Coordinated Expression across the F_2_ Mice The rescaled expression levels of 184 distinct transcripts (y-axis) that belong to the lipid metabolism pathway are shown for each of the 60 F_2_ mice (x-axis). Plot shows that the subset of lipid metabolism traits identified by our trait correlation-GO analysis has preserved vertical patterns (*P* = 4e10^−4^ by permutation test).

The strategy given above can be applied to many metabolic pathways. We found evidence of a regulatory pathway controlling lipid metabolism (Figure 4; *P* = 4e10^−4^). These expression patterns were combined from lists using 71 different seeds. As was the case for the GPCR transcripts, these show evidence of coregulation across the 60 F_2_ mice.

We have previously studied eQTL for stearoyl-CoA desaturase-1 *(Scd1)* [2], an important gene for lipid metabolism and insulin sensitivity [18,19]. When we used *Scd1* expression as a seed, the traits that were most closely correlated with *Scd1* were clearly enriched in lipid metabolism genes (Table S5).

The traits that are highly correlated also map to the same genomic locations, even though their respective genes are in separate locations (i.e., they are being regulated in *trans*). Major QTL peaks for most of the 20 lipid metabolism traits were found on Chromosomes 2 *(D2Mit263)* and 5 *(D5Mit240);* to a lesser extent, they were found by loci on several other chromosomes. We found strong evidence that these two loci are jointly predictive for all 20 traits (Table 1).

**Table 1:**
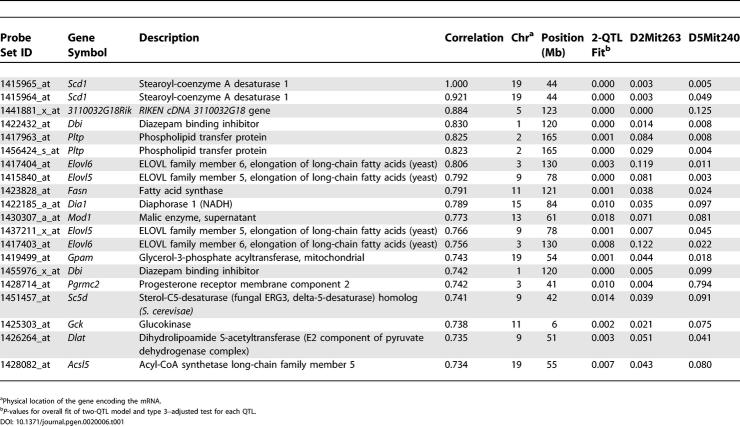
Top 20 Gene Expression Traits Correlated with *Scd1* Are All Lipid Metabolism Genes

Two genes that ranked high in Table 1, *RIKEN cDNA 3110032G18* (*Riken32G18* hereafter; *r* = 0.868) and diazepam binding inhibitor (*r* = 0.816), did not seem to have obvious functional relevance with *Scd1.* We used this opportunity to test the predictive power of trait correlations.

### Diazepam Binding Inhibitor Is Functionally Related to *Scd1*

The correlation between diazepam binding inhibitor *(Dbi)* and *Scd1* is stronger than that for the classic lipid biosynthesis genes such as fatty acid synthase, malic enzyme, and fatty acyl-CoA ligase. Interval mapping of the expression levels of *Dbi* and *Scd1* showed highly similar patterns (data not shown), suggesting that they are regulated by common loci. To understand the biological basis for the predicted coregulation, we first sought to use in silico approaches to investigate this gene. The *Dbi* gene symbol is synonymous with acyl-CoA binding protein *(Acbp).* ACBP is a 10-kDa cytosolic protein that binds long-chain fatty acyl-CoAs and has a high affinity for oleoyl-CoA, the product of the SCD1 reaction [20]. Our data analysis predicts that a common regulator maintains a fixed ratio of mRNA for SCD1 and for a protein that binds its product, oleoyl-CoA. Recently, *Dbi* was reported to be regulated by sterol regulatory element binding protein-1c (SREBP-1c), a known master regulator of lipogenic enzymes [21]. Perhaps one or more of the regulators we have mapped are coactivators or corepressors of SREBP-1c, analogous to PPARγ coactivator-1β [22]. Thus, the prediction from our trait correlation analysis that *Dbi* is functionally related to *Scd1* was confirmed by recently published experimental data.

### Prediction of *Riken32G18* as a Putative Lipid Metabolism Gene

The mRNA most closely correlated to *Scd1* is *Riken32G18,* which maps to the same location on Chromosome 2 as *Scd1* expression but with almost twice the LOD score. Notably, *Riken32G18* is physically located on Chromosome 5 and *Scd1* is on Chromosome 19; thus, the two genes are likely coregulated in *trans* by the Chromosome 2 locus.

We hypothesized that *Riken32G18* plays a role in lipid metabolism with a close functional relationship with *Scd1.* There is no information available from the online resources to functionally link *Riken32G18* to *Scd1* or to lipid metabolism. In order to test its functional relationship with lipid metabolism, we asked if *Riken32G18* expression is responsive to conditions that regulate expression of lipid metabolism genes.

**Figure 5:**
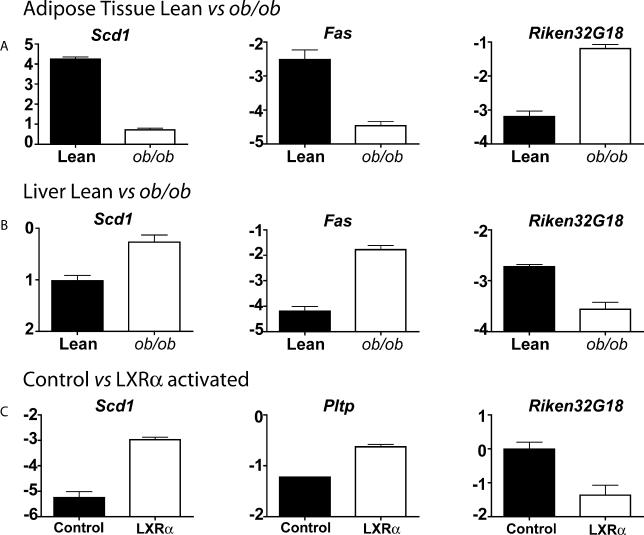
Regulation of *Riken32G18* Inversely Parallels the Regulation of Lipogenic Genes (A) Metabolic regulation of *Riken32G18*. Expression of lipogenic genes and *Riken32G18* in adipose tissues of lean versus obese mice. (B) Expression of lipogenic genes and *Riken32G18* in livers of lean versus obese mice. (C) Expression of LXRα target genes and *Riken32G18* in mouse livers treated with LXRα agonist T0901317.

From our previous results, we know that lipogenic genes are expressed at a lower level in *ob/ob* adipose tissue than in lean adipose tissue [23]. Whereas *Scd1* and fatty acid synthase *(Fas)* were expressed at a lower level in *ob/ob* adipose tissue, *Riken32G18* expression was higher in obese adipose tissue. Thus, *Riken32G18* appeared to be regulated reciprocally with lipogenic genes (Figure 5A). In contrast to adipose tissue, lipogenic genes are more highly expressed in livers of *ob/ob* mice than in lean mice [24]. Again, *Riken32G18* was regulated in the opposite direction; i.e., its expression was reduced in *ob/ob* liver (Figure 5B). Finally, liver-X-receptor (LXR) α activation increases genes in fatty acid metabolism in the liver. Once again, *Riken32G18* expression was down-regulated by LXRα activation (Figure 5C). We conclude that *Riken32G18* is very likely a novel gene involved in lipid metabolism. It is coregulated with lipid metabolism genes across the 60 F_2_ mice and reciprocally regulated with such genes across physiologic conditions.

## Discussion

Macromolecules and metabolites in cells interact with one another across a critical concentration range. The functional concentration must be within boundaries defined by the kinetics and thermodynamics of these interactions. One way cells can maintain the critical concentration range for molecules within a pathway is to maintain them under the control of a common regulatory system. A well-characterized example is the regulation of lipogenic enzymes by SREBP-1c, a master regulator of virtually all enzymes critical to fatty acid synthesis [25]. Pathways that feed into lipogenesis are also coregulated with lipogenic enzymes.

Whereas Eisen et al. [4] studied gene expression correlation over a time dimension and Zhang et al. [3] studied gene expression correlation over a tissue dimension, we performed our studies over a genetic dimension. In addition to identifying coregulated genes, the genetic dimension also supplies us with information about the genomic location of putative regulatory loci. Our method involved a two-step approach. First, we applied a genomewide linkage analysis to identify mapping transcripts using a liberal filter to ensure a high true-positive rate. In the second step, we performed trait correlation analysis, using seeds within linkage groups.

Our approach revealed biologically meaningful relationships among the traits. In particular, we found that traits that are correlated over a genetic dimension are frequently enriched for a physiologically meaningful biological function. For example, we found a gene whose expression was highly correlated with those of lipid metabolism genes and with *Scd1* expression as a seed trait. The prediction that this gene is involved in lipid metabolism was borne out by recent studies showing that this protein binds to oleoyl-CoA, the product of the SCD1 reaction. Moreover, the eQTL for this gene maps to the same location on distal Chromosome 2 as did other lipid metabolism eQTL.

The distal region of Chromosome 2 containing the cluster of lipid metabolism traits is quite interesting as several QTL for obesity and related traits have been mapped to this region [13,26–30]. Furthermore, Estrada-Smith et al. [31] have used congenic mouse lines derived from B6 and CAST/Ei strains to identify a 17-Mb region of distal Chromosome 2 that regulates plasma levels of triglycerides. This region directly overlaps with the location of our fatty acid metabolism cluster and our fasting plasma insulin locus.

We also found an entirely unannotated gene highly correlated with lipid metabolism genes. Analysis of tissues from obese animals or animals treated with an LXRα agonist revealed that this gene is regulated inversely under these physiologic conditions with lipid metabolism genes, providing evidence that it participates in lipid metabolism or perhaps is a negative regulator of lipid metabolism.

Another example of coordinated expression was the GPCR protein signaling pathway genes. Here, we found that *all* of the 174 transcripts that were highly correlated with 38 GPCR seeds encoded GPCR pathway proteins and most mapped to the same locus on Chromosome 2. Fewer than 10% of the transcripts are olfactory receptors. Among the other correlating genes are gamma-aminobutyric acid (GABA) receptor signaling pathway genes, neuropeptide signaling pathway genes, and cAMP signaling genes (Table S6).

The GPCR protein signaling pathway cluster overlaps with a region on proximal Chromosome 2 that was narrowed down to an 8-Mb region regulating body weight differences in the B6 and BTBR strains [32] and a 10-Mb region underlying obesity-related traits in the B6 and CAST/Ei strains [31]. Several of the GPCRs in the cluster are related to obesity traits in mice when mutated or overexpressed [33–36], and others have been proposed as candidate genes for human obesity [37–39] (Figure S3A). An additional subset of the GPCR cluster represents GABA receptor subunits (Figure S3B). Alterations in the GABA system are related to the obesity phenotype seen in obese *(fa/fa)* Zucker rats [40] and humans with Prader-Willi syndrome [41,42].

Although we have focused our own analysis on lipid metabolism, our database, which is available through WebQTL (www.genenetwork.org) [43], can provide insights into the regulation of metabolic pathways expressed in the liver. A researcher can start with a particular mRNA of interest as a seed trait and quickly identify genes whose expression is highly correlated with the seed trait. By looking for overlapping loci that control the expression of the seed and additional genes of interest, a regulatory pathway can be hypothesized.

The particular implementation of the approach can and should be study specific. For example, there are a number of thresholds that must be determined, each affecting the number of transcripts considered and the overall FDR. The level of tolerable FDR depends on a number of factors. We accepted a very high FDR in our first stage of transcript mapping as we desired only a mild filtering of the data to reduce the computational burden. We have recently developed statistical methods that provide better control of FDR for eQTL mapping [17]. These could be used if a more stringent filtering was desired at this stage. A more conservative filtering in the second stage was used here by imposing a relatively high threshold for the correlation coefficient. Although a correlation coefficient threshold as low as 0.4 would still correspond to an acceptable FDR of 0.05, we chose the more conservative 0.7. We have reported stage-specific FDRs as they were used to guide our analysis. However, we note that the FDR associated with the entire procedure is not known. Indeed, it is an extremely difficult problem to precisely define, not to mention determine, overall FDR for studies involving multiple types of data and multiple analysis methods. Doing so remains an open question of importance to both statisticians and biologists.

In summary, here we used two complementary approaches. First, we started with single QTL mapping to obtain seeds. Second, we used the seeds to obtain correlated traits. Finally, we confirmed genetic architecture for the correlated traits. As we have argued, the combined approach is more sensitive to retrieve biologically meaningful information from the data set. If we just did QTL mapping, those traits with nonsignificant LOD scores would have been missed. If we just performed correlation analysis, we would have lost the causation information that comes from the genetic linkage analysis. Our approach provides an important organizing step in the analysis of expression networks. It yields sets of related traits that can be further analyzed using multiple trait mapping [44] and causal networks [45], as these methods are most effective after screening to obtain a modest number of closely related traits. Moreover, our approach increases the power to formally uncover members of gene expression networks.

## Materials and Methods

### Animals.

The power of a genetic mapping study depends on the heritability of the trait, the number of individuals included in the analysis, and the genetic dissimilarity among them. In this study, we selected 60 (B6 × BTBR) F_2_-*ob/ob* mice (29 males and 31 females) based on the selective phenotyping algorithm that we developed [14]. The algorithm selects a subset of F_2_ individuals from a mapping panel based on genotype data. The selection achieves substantial improvements in sensitivity compared to a random sample of the same size [14]. The 60 mice used in this study were a subset of F_2_ mice segregating for phenotypes associated with obesity and diabetes [13]. The F_1_ mice used to generate the F_2_ sample were all derived from crosses in which the B6 parent was male and the BTBR parent was female. The framework map consists of 194 microsatellite markers, with an average spacing of 10 cM.

### Microarrays.

Liver total RNA was extracted from frozen tissue samples with RNAzol reagent (Tel-Test, Friendswood, Texas, United States). Crude RNA samples were purified with RNeasy mini-columns (Qiagen, Valencia, California, United States) before being subjected to microarray and RT-PCR studies. The RNA samples were processed according to the Affymetrix Expression Analysis Technical Manual. A total of 60 MOE430A and MOE430B arrays were used to monitor the expression levels of approximately 45,000 genes or ESTs. The data were processed with MAS5.0 to generate cell intensity files. Quantitative expression levels of all the transcripts were estimated using the RMA algorithm for normalization [15].

### Identifying transcripts with linkages.

To identify traits that have linkages in the 60 F_2_ mice, we used standard interval mapping implemented in R/qtl [46] to map each of the 45,037 unique probe sets at 5-cM resolution. Transcripts with LOD scores of 3.4 or greater at one or more locations in the genome were recorded as transcripts with linkages, or “seeds.” Using maximum LOD of 3.4 or greater as a threshold roughly corresponds to genomewide type I error rate of 0.08 [16]. We permuted the animal labels five times and for each permutation ran R/qtl interval mapping on a 5-cM grid. The loci-specific LOD scores from the five permutations were pooled together to estimate empirical *P-*values These *P-*values were then used for calculation of q-values and estimation of FDR [47]. The FDR corresponding to a LOD score of 3.4 was 0.48. This resulted in 6,016 mapping traits. We found some regions of the genome with strong sex-by-genotype interactions. The results reported here are in regions with little or no evidence for such interactions.

### Hierarchical clustering of the LOD score profiles.

LOD score profiles were obtained for each transcript using R/qtl interval mapping with 5-cM intervals. This yielded, for each trait, 465 interval mapping testing points in the entire genome. Treating each LOD score profile as a point in the 465-dimensional space, we calculated pairwise Euclidean distances between LOD score profiles and performed hierarchical clustering based on those distances. Clustering the LOD profiles has the advantage that transcripts having similar mapping patterns across the genome will be grouped together.

### Expression correlation.

Using each of the 6,016 transcripts as a seed, Pearson correlation coefficients were calculated against the log-transformed expression intensities (raw intensities were normalized by RMA) of all the 45,037 transcripts in 60 F_2_ mice. We obtained a 6,016-by-45,037 matrix of correlation coefficients. For each seed, we collected the traits with a correlation coefficient of 0.7 or greater, which forms the list of correlated traits for that seed. For each pair of transcripts, we computed its Pearson correlation together with the *P-*value. We then computed the corresponding q-values to determine the FDR [47]. A correlation of 0.7 corresponds to an FDR of 1.52e10^−6^.

### Test for enrichment.

Enriched functional groups, defined as GO categories corresponding to BP, were identified from each list with *P-*values computed from a Hypergeometric calculation, carried out using a custom variant of “GOHyperG” in the “GOstats” package (version 1.1.3; Bioconductor Core Team 2004; http://www.bioconductor.org). Interpretation of these *P-*values is not straightforward since many dependent hypotheses are tested. Furthermore, the Hypergeometric calculation tends to result in small *P-*values when groups with few transcripts are considered. It has been suggested that one consider only GO nodes with small *P-*values and a reasonable number of genes (ten or more) [48]. For each list, we examined up to 50 GO terms, each of which involves more than ten genes and has a Hypergeometric *P-*value of ≤0.01.

### Creating the coordinated expression plot.

From the lists of correlated traits, we identified 15,726 BP GO terms in total, among which 862 are unique. For each of the unique GO terms, we traced back the lists of correlated traits that were enriched (*P* = 0.01), and we concatenated all such lists. To create the heat plot, we first normalized the log intensity values of each trait across all the 60 F_2_ mice to mean = 0 and variance = 1. This step is very important because some traits were highly overexpressed or underexpressed across all the F_2_ mice. We then plotted the rescaled expression values of transcripts across the 60 F_2_ mice.

### Permutation test for coordinated expression.

We developed a statistic to assess the extent of coordinate expression. We first collapsed all the members in a list into a pseudo-member by taking the average across the transcripts in that list. We then computed pairwise correlations among the pseudo-members. The final measure of coordinate expression was the average of those pairwise correlations. For a given GO term, we sampled the same number of lists from the total of 1,341 lists 10,000 times and matched the list sizes to those in the GO term of consideration. We computed the statistic in each random sample. The empirical *P*-value was calculated as the frequency of the samples where the statistic exceeded the one calculated using the observed data. The *P-*value for GPCR protein signaling pathway set was 1e10^−4^, and it was 4e10^−4^ for the lipid metabolism set.

### Genetic architecture of GO groups.

Subsequent QTL analyses were performed on traits within GO groups using one- and two-QTL scans with R/qtl. Possible interacting effects of sex were examined and found not to be significant for the GPCR and lipid metabolism groups reported here (data not shown).

### Real-time quantitative PCR for *Riken32G18* and lipogenic genes.

First-strand cDNA was synthesized from 1 μg of total RNA using Super Script II Reverse Transcriptase (GIBCO BRL, San Diego, California, United States) primed with a mixture of oligo-dT and random hexamers. Reactions lacking the reverse transcriptase served as a control for amplification of genomic DNA. Representative genes encoding enzymes in lipid metabolism pathways were studied together with *Riken32G18,* including *Scd1, Fas,* and phospholipid transfer protein *(Pltp).* The housekeeping gene β*-actin* was used as a normalization control.

### LXRα agonist treatment of mice.

BTBR male 7-wk-old mice were fed a chow diet (Harlan 7001 4% fat rodent chow) supplemented with DMSO vehicle or DMSO + T0901317 (0.025% by weight; Tularik, San Francisco, California, United States) for 7 d. T0901317 is a nonsteroidal LXR agonist that has previously been shown to potently transactivate LXR-regulated genes in mice [49]. After a 4-h fast, liver samples were collected and frozen in liquid nitrogen.

## Supporting Information

Figure S1 LOD Profiles of ATP Synthase Subunit mRNA Traits The traits exhibiting a linkage peak on Chromosome 2 are grouped together on top rows.
(477 KB PDF)

Figure S2 LOD Profiles of 38 Seed Traits That Produce the 174 Correlated Traits Belonging to the GPCR Pathway (495 KB PDF)

Figure S3 LOD Profiles of GPCR eQTL (A) LOD profiles of GPCR protein signaling pathway mRNA traits related to obesity. The LOD profiles were generated on WebQTL. The empirical *P-*values are generated by permutation test (1,000 permutations); the lower line indicates suggestive linkage and the upper line indicates significant linkage. The green line indicates BTBR alleles increase the trait values; the red line indicates that B6 alleles increase the trait values.
(B) LOD profiles of GPCR protein signaling pathway mRNA traits related to obesity, from the GABA receptor subunit family. The LOD profiles were generated on WebQTL. The empirical *P*-values are generated by permutation test (1,000 permutations); the lower line indicates suggestive linkage and the upper line indicates significant linkage. The green line indicates BTBR alleles increase the trait values; the red line indicates that B6 alleles increase the trait values.
(3.2 MB PDF)

Table S1 *cis* Traits Inferred by Using LOD Greater Than 3.4 Columns A, B, and C show the Affymetrix probe set IDs, gene names, and the gene symbols of the *cis* traits. Columns D and E show the physical chromosome and position (Mb) of the traits. Column F shows the maximum LOD scores of the *cis* traits.
(46 KB TXT)

Table S2 Enriched GO Categories for Expression Traits That Are Correlated with the Seed Traits Using each of the 6,016 transcripts as a seed, Pearson correlation coefficients were calculated against the log-transformed expression intensities (raw intensities were normalized by RMA) of all of the 45,037 transcripts in the 60 F_2_ mice. For each seed, we collected the traits with correlation coefficients of 0.7 or greater, which form the list of correlated traits for that seed. Enriched functional groups, defined as GO categories corresponding to BP, were identified from each list with *P-*values computed using a custom variant of “GOHyperG” in the “GOstats” package (Bioconductor Core Team 2004; http://www.bioconductor.org). For each list, we examined up to 50 GO terms, each of which involves more than ten genes and has a *P*-value of ≤0.01. Column A contains the Affymetrix probe set identifiers. Column B contains the names of the genes. Columns C through AZ are the GO terms identified, and Columns BA through CX are the identifiers of these GO terms.
(948 KB TXT)

Table S3 GO Enrichments of Traits That Are Correlated with Seed Traits From the lists of traits correlated with the seed traits in Table S2, we identified 15,726 BP GO terms in total, among which 862 are unique. For each of the unique GO terms, we traced back the lists of correlated traits that were enriched (*P* = 0.01), and we concatenated all such lists. Columns A and B show the GO category identifiers and the names, respectively. Columns C and D show the probe set IDs and gene names of the correlated traits. Column E shows whether (“T” or “F”) the traits were annotated as belonging to the corresponding GO terms in the public database.
(8.3 MB TXT)

Table S4 Descriptions of the 174 GPCR Protein Signaling Pathway Traits Columns A, B, and C show the Affymetrix probe set IDs, gene symbols, and gene titles of the 174 gene traits. Columns D and E show the physical chromosomes and positions (Mb) of the 174 traits. Columns F, G, and H show the chromosomes, positions (Mb), and LOD scores of the maximum LOD peaks of those 174 traits.
(12 KB TXT)

Table S5 Top 100 Traits Whose Expression Levels Are Correlated with Those of *Scd1* in the 60 F_2_ Mice Column A shows the Affymetrix probe set IDs. Columns B and C show the gene symbols and the titles of the genes. Columns D and E show the genomic locations of these traits. Column F shows the Pearson correlation coefficients. Column G shows the unadjusted *P-*values for the correlations.
(7 KB TXT)

Table S6 Descriptions of the Gene Ontology “BP” Terms That Are Enriched in the 174 GPCR Protein Signaling Pathway Traits Columns A and B show the GO category identifiers and the names. Column C shows the enrichment *P-*values (rounded to five decimal places) calculated using Hypergeometric distribution. Column D shows the GO category size.
(1 KB TXT)

### Accession Numbers

The original genotypes, physiologic phenotypes, and microarray-derived gene expression data are available in WebQTL (http://www.genenetwork.org; group: B6BTBRF2 [43]) and in GEO (http://www.ncbi.nlm.nih.gov/geo; accession number GSE3330).

The Gene Ontology (www.geneontology.org) accession numbers are GPCR protein signaling pathway (0007186) and *Scd1* (1415965_at).

## Abbreviations

BP: Biological Process
eQTL: expression quantitative trait loci
FDR: false discovery rate
GABA: gamma-aminobutyric acid
GO: Gene Ontology
GPCR: G protein–coupled receptor
LXR: liver-X-receptor
RMA: Robust Multi-array Average
