# Supplementary material for: Combined Expression Trait Correlations and Expression Quantitative Trait Locus Mapping
Source: PLoS Genet. 2006 Jan 20;2(1):e6. doi: 10.1371/journal.pgen.0020006 (PMC1331977; doi:10.1371/journal.pgen.0020006)
Supplement: Supplementary file 1 [file pgen.0020006.sg001.pdf]

Figure S1

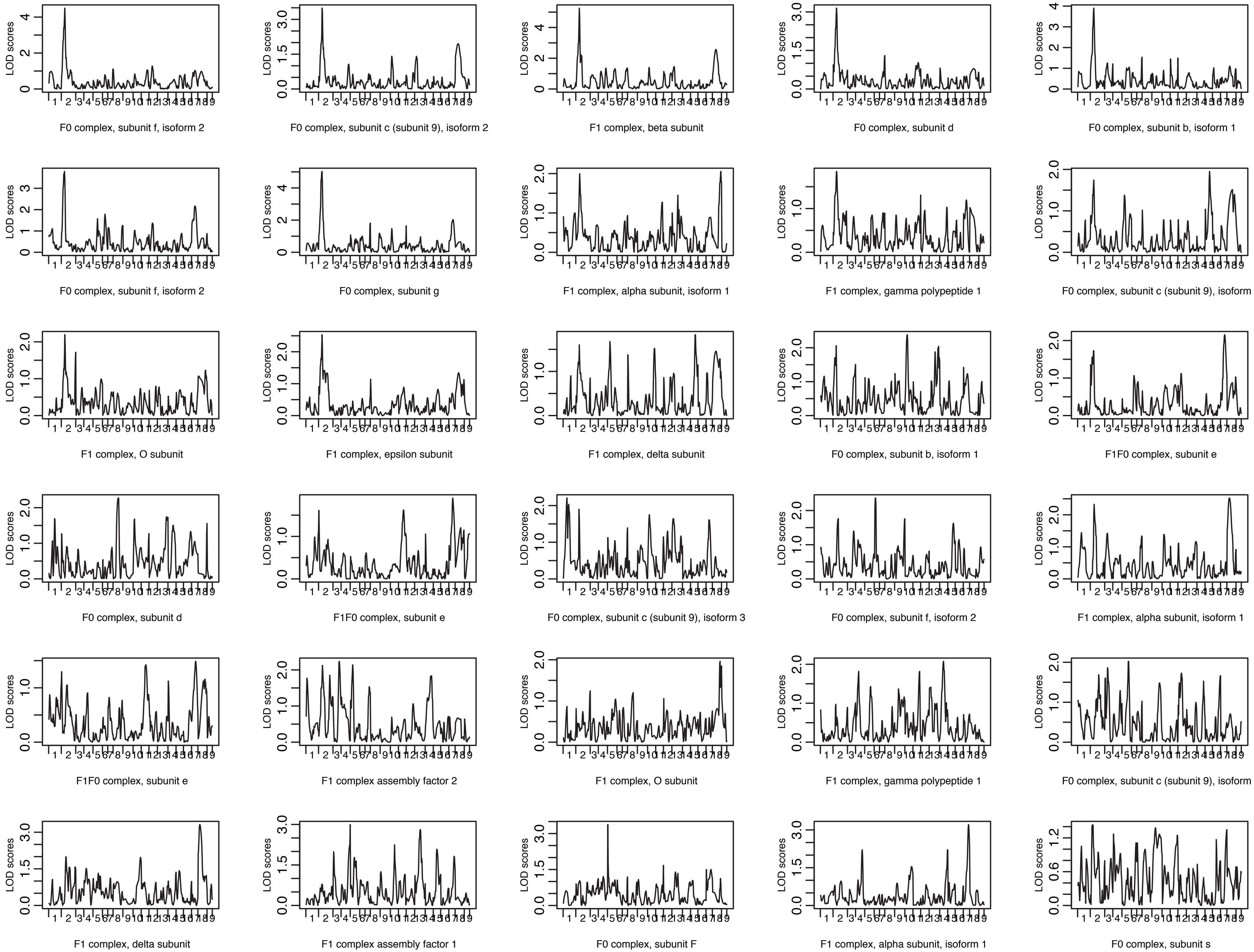
LOD scores
F0 complex, subunit f, isoform 2
F0 complex, subunit c (subunit 9), isoform 2
F1 complex, beta subunit
F0 complex, subunit d
F0 complex, subunit b, isoform 1
F0 complex, subunit f, isoform 2
F0 complex, subunit g
F1 complex, alpha subunit, isoform 1
F1 complex, gamma polypeptide 1
F0 complex, subunit c (subunit 9), isoform 1
F1 complex, O subunit
F1 complex, epsilon subunit
F1 complex, delta subunit
F0 complex, subunit b, isoform 1
F1F0 complex, subunit e
F0 complex, subunit d
F1F0 complex, subunit e
F0 complex, subunit c (subunit 9), isoform 3
F0 complex, subunit f, isoform 2
F1 complex, alpha subunit, isoform 1
F1F0 complex, subunit e
F1 complex assembly factor 2
F1 complex, O subunit
F1 complex, gamma polypeptide 1
F0 complex, subunit c (subunit 9), isoform 2
F1 complex, delta subunit
F1 complex assembly factor 1
F0 complex, subunit F
F1 complex, alpha subunit, isoform 1
F0 complex, subunit s
