# Supplementary material for: Combined Expression Trait Correlations and Expression Quantitative Trait Locus Mapping
Source: PLoS Genet. 2006 Jan 20;2(1):e6. doi: 10.1371/journal.pgen.0020006 (PMC1331977; doi:10.1371/journal.pgen.0020006)
Supplement: Supplementary file 2 [file pgen.0020006.sg002.pdf]

Figure S2

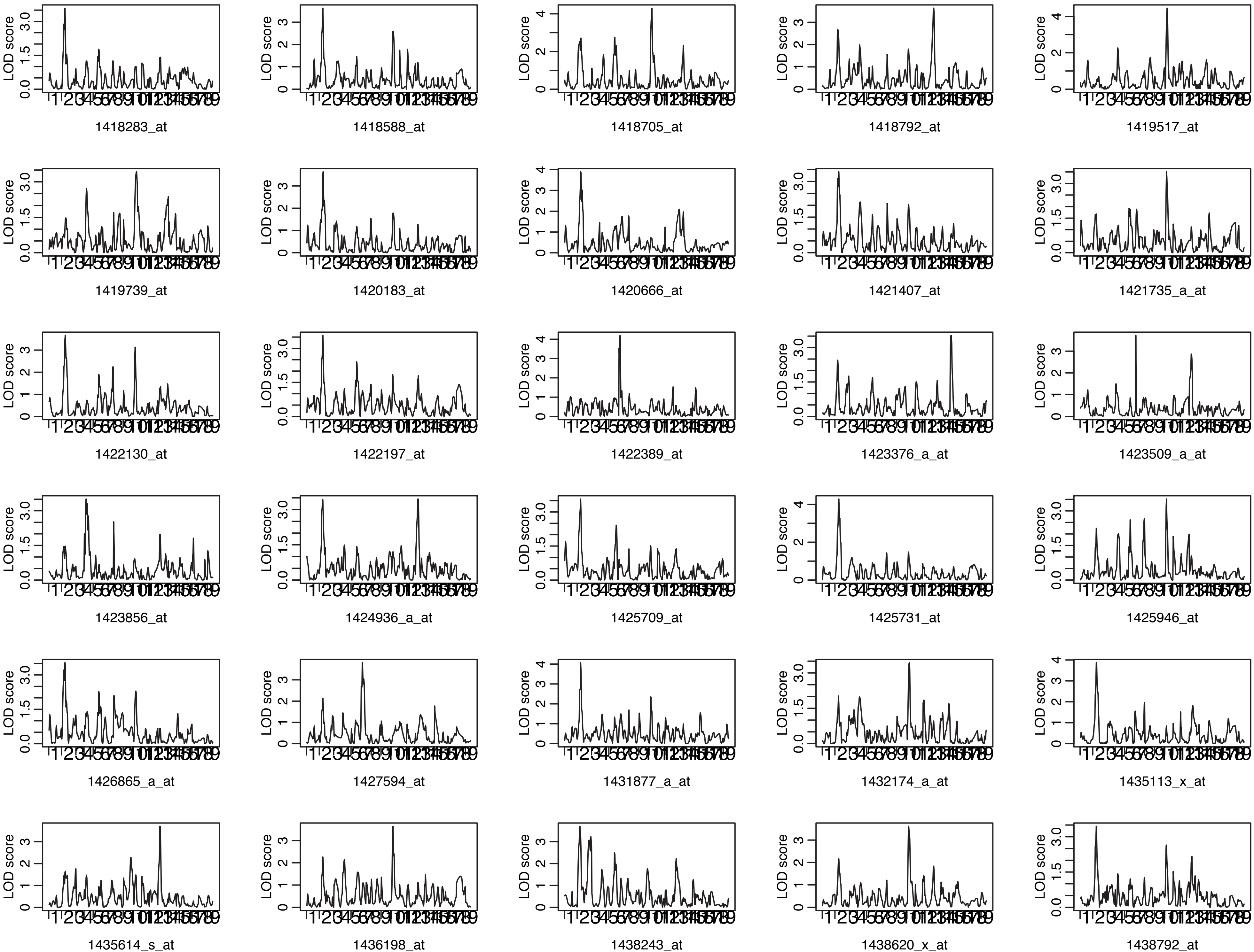
LOD score
1418283_at
1418588_at
1418705_at
1418792_at
1419517_at
1419739_at
1420183_at
1420666_at
1421407_at
1421735_a_at
1422130_at
1422197_at
1422389_at
1423376_a_at
1423509_a_at
1423856_at
1424936_a_at
1425709_at
1425731_at
1425946_at
1426865_a_at
1427594_at
1431877_a_at
1432174_a_at
1435113_x_at
1435614_s_at
1436198_at
1438243_at
1438620_x_at
1438792_at

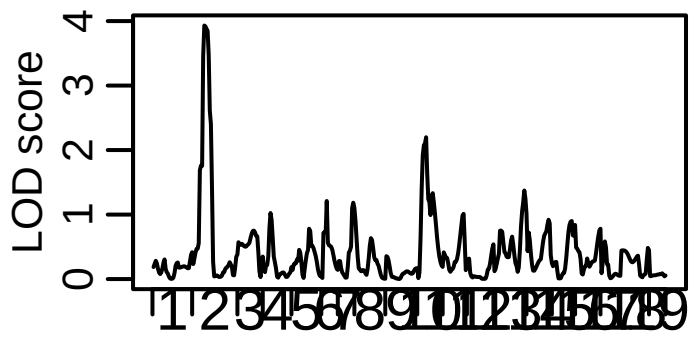

1448299_at

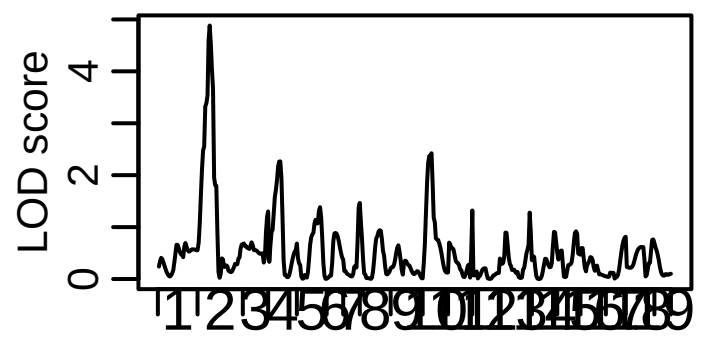

1448628_at

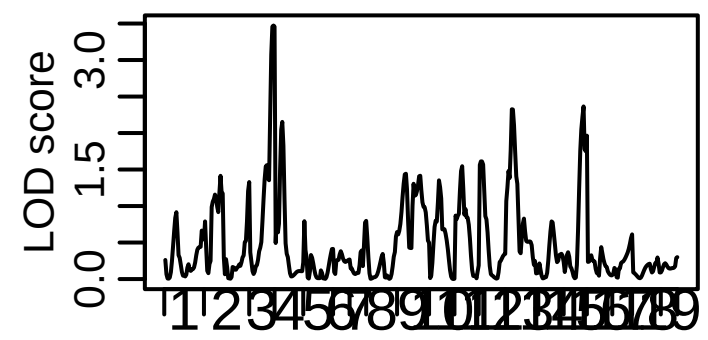

1449464_at

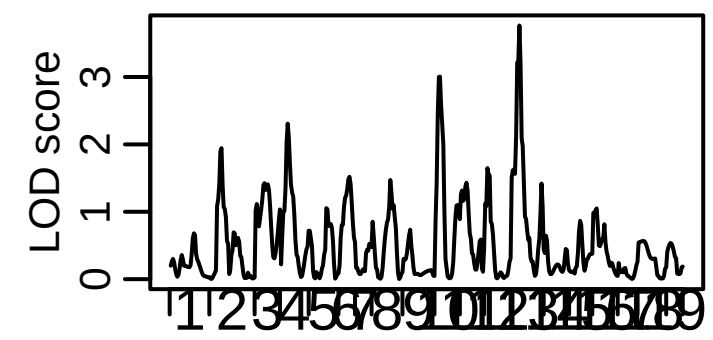

1449995_at

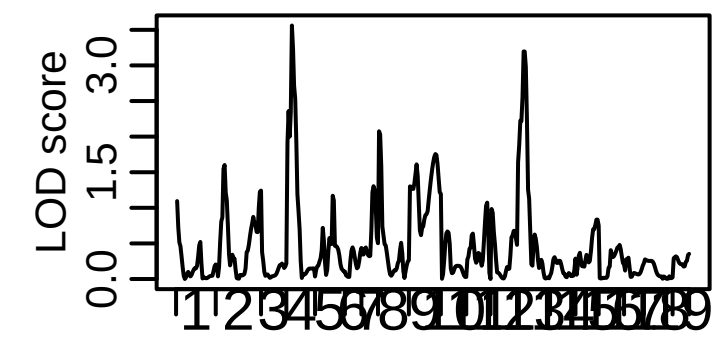

1450513_at

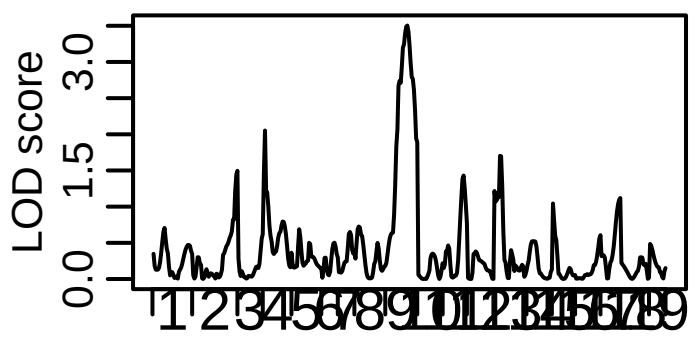

1455825_s_at

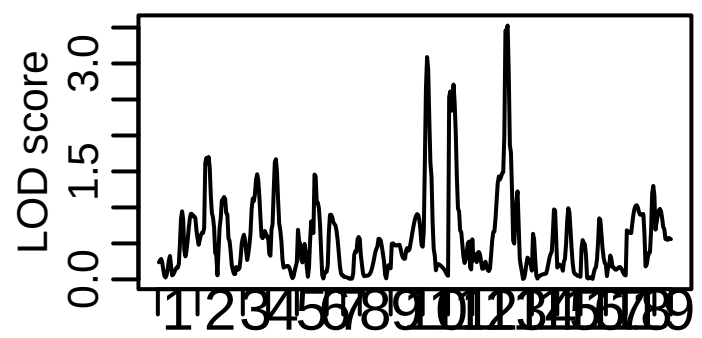

1457521_at

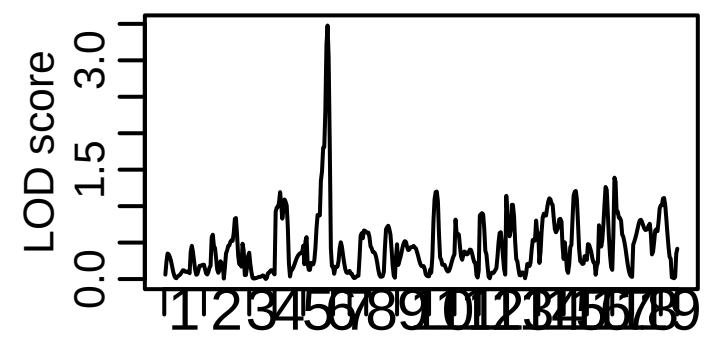

1457594_at
