# Supplementary material for: Combined Expression Trait Correlations and Expression Quantitative Trait Locus Mapping
Source: PLoS Genet. 2006 Jan 20;2(1):e6. doi: 10.1371/journal.pgen.0020006 (PMC1331977; doi:10.1371/journal.pgen.0020006)

Figure S3A

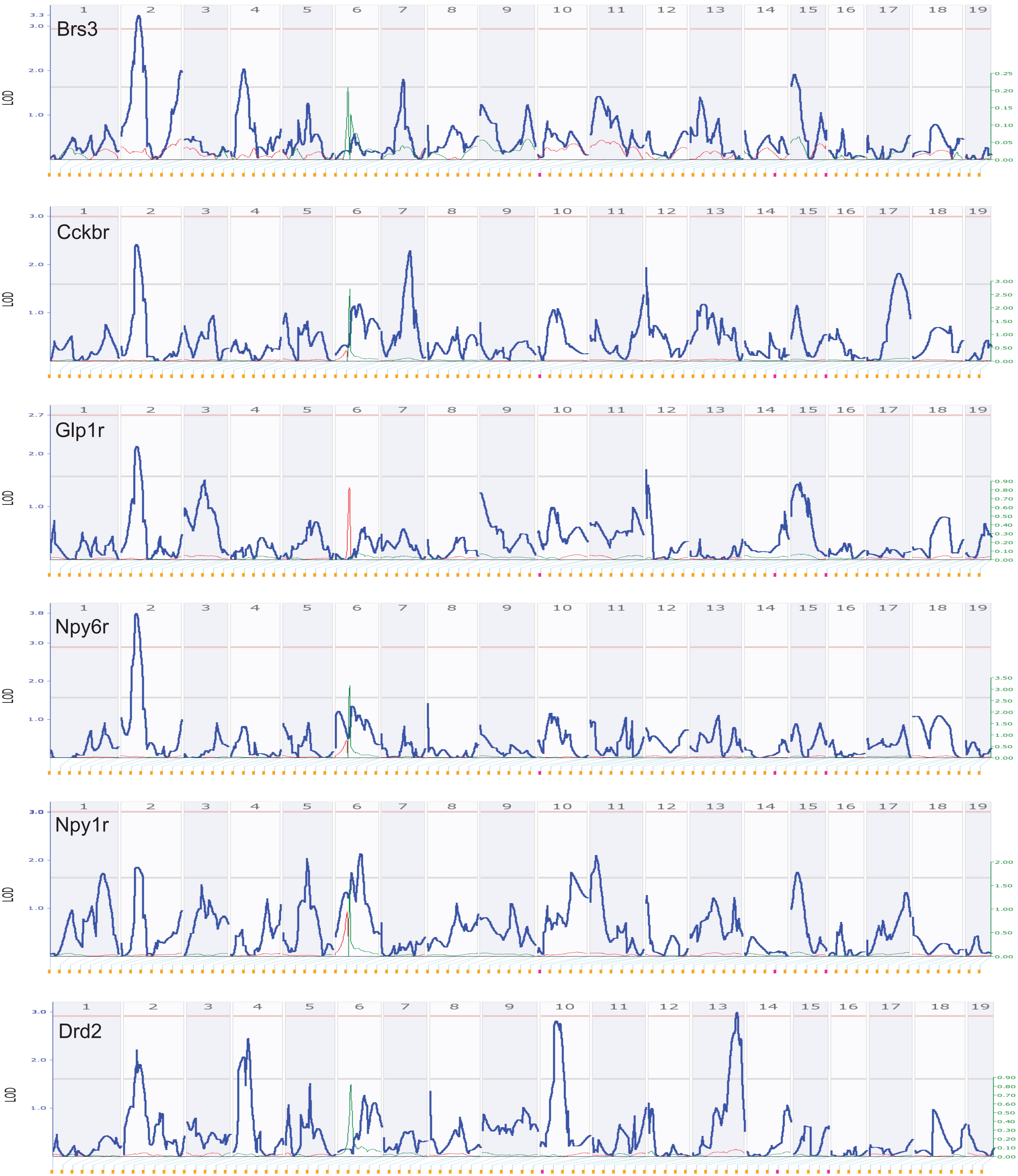

Brs3
Cckbr
Glp1r
Npy6r
Npy1r
Drd2
LOD
1 2 3 4 5 6 7 8 9 10 11 12 13 14 15 16 17 18 19

Figure S3B

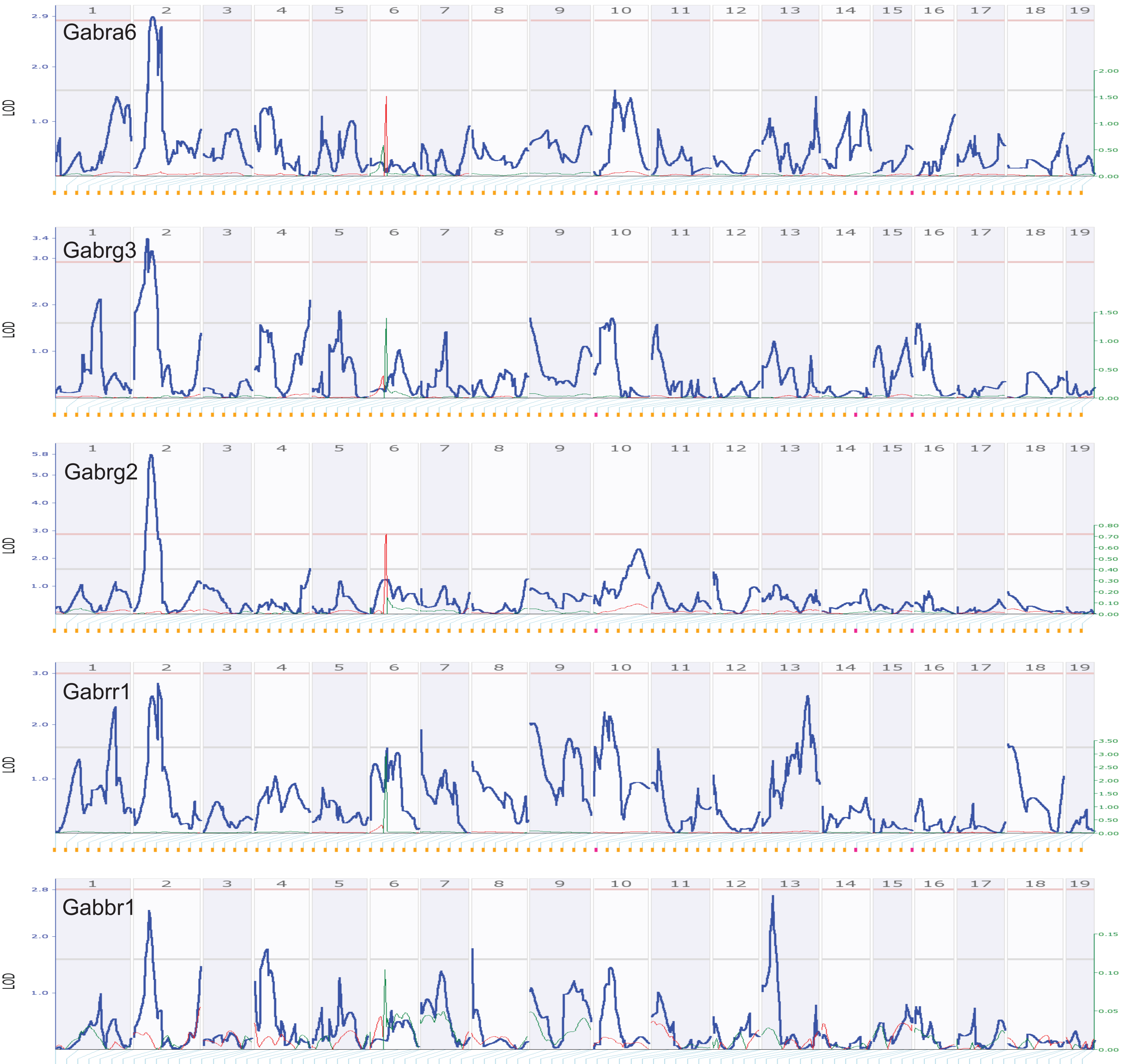

Supplement: Supplementary file 3 [file pgen.0020006.sg003.pdf]
